# Delayed onset purpura following periorbital vessel removal with a long-pulsed Nd:YAG laser

**DOI:** 10.1016/j.jdcr.2025.01.002

**Published:** 2025-01-13

**Authors:** Samar Khalil, Pia Maria Obeid, Hassan Galadari, Omar A. Ibrahimi

**Affiliations:** a Skin & Scalpel Clinic, Sin el Fil, Lebanon; b Lebanese American University Medical Center–Rizk Hospital, Beirut, Lebanon; c Department of Internal Medicine, College of Medicine and Health Sciences, United Arab Emirates University, Al Ain, United Arab Emirates; d Connecticut Skin Institute, Stamford, Connecticut

**Keywords:** bruising, Nd:YAG, peri-orbital veins, purpura, vascular laser

## Introduction

The skin around the eyes is the thinnest, making it especially prone to early signs of aging. Skin laxity, wrinkles, dark circles, periorbital vessels, and pigmentation are frequent complaints among patients.^1^ Minimally invasive procedures, including various topicals, fillers, botulinum toxin, lasers, radiofrequency devices, and chemical peels, can be used to rejuvenate the periorbital region.^2^

A common complaint of patients is the presence of prominent periorbital vessels.^3^ Treatments such as sclerotherapy, surgical excision, and electrosurgery may be employed, but given the proximity to critical structures and possible complications, these modalities are used sparingly nowadays.^4^

The theory of selective photothermolysis allows the use of vascular specific lasers as a safe and effective treatment option.^5^ Generally, periorbital vessels can be targeted using the pulsed dye laser, potassium titanyl phosphate laser, and neodymium-doped yttrium aluminum garnet (Nd:YAG) laser to target hemoglobin in red blood cells. The desired clinical endpoint is vessel disappearance or a temporary graying/darkening of these vessels.^6^ While these lasers are increasingly recognized for effectively treating periorbital veins, more research is needed to fully establish their safety and develop guidelines for their use and aftercare in this specific area.^7^^,^^8^

One of the most important considerations when treating periorbital veins is proper eye protection using ocular shields. Skin cooling is essential to minimize thermal damage of unintended chromophores. However, excessive cooling can cause vasoconstriction, potentially reducing the effectiveness of the treatment. Typically, 1-3 treatment sessions are needed to achieve optimal results, with at least 4-6 weeks between sessions. Some patients may experience mild discomfort, redness, swelling, or immediate purpura following treatment.^7^

Here, we report for the first time, a case series of a previously unreported adverse side effect of delayed onset purpura occurring months following treatment of periorbital vessels with a 1064 nm vascular laser.

## Case report

Patients underwent a session of periorbital vessel treatment using a 1064 nm long pulsed Nd:YAG laser (The R35-NX handpiece of Fotona NX Dynamis was used in cases 1, 2, and 4. Cutera Excel V was used in case 3). A cold ultrasound gel was applied before the procedure. The laser settings employed were as follows: energy of 140 J/cm^2^, pulse duration of 25 milliseconds, and spot size of 4 mm. In cases 1, 3 and 4, only the upper eyelids were treated. In case 2, both the upper and lower eyelids were treated.

### Case 1

**Fig 1:**
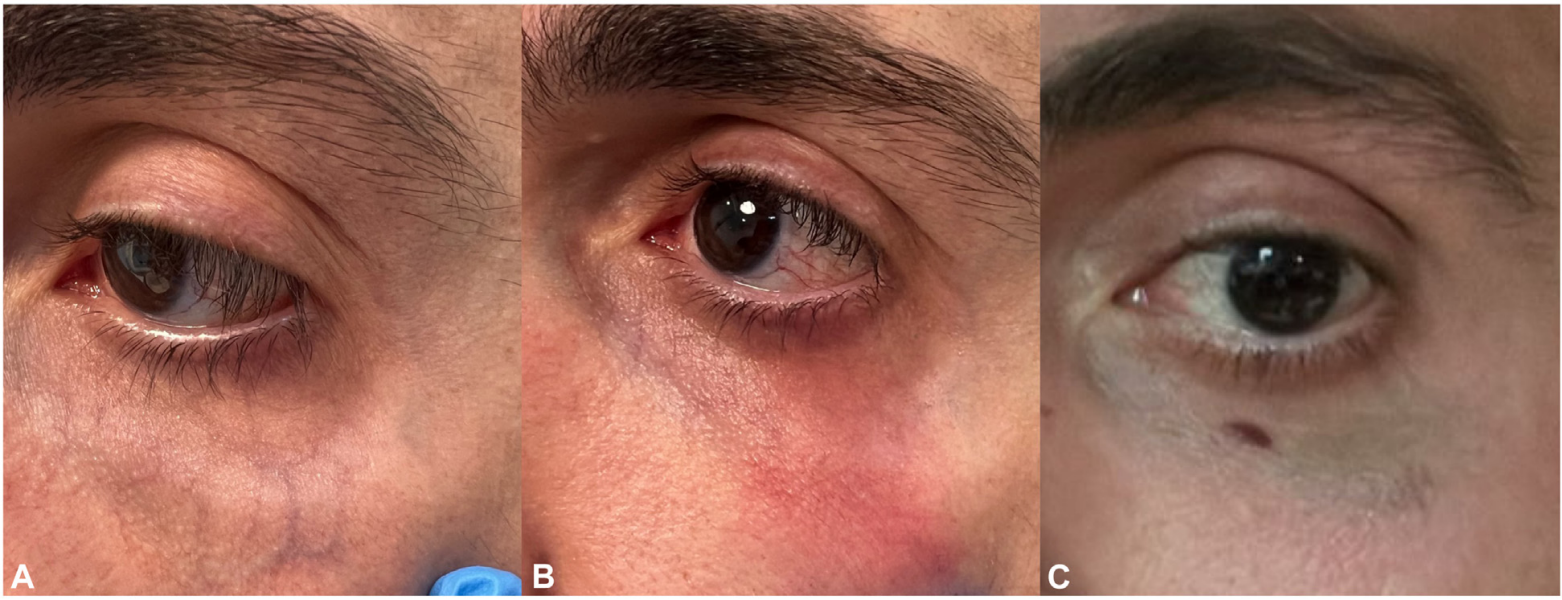
**A,** Before, (**B**) Immediately after, and (**C**) 2 months after the laser procedure.

A 35-year-old healthy Lebanese male experienced bruising in his left lower eyelid 2 months after the treatment (Fig 1). He denied any medications/supplements or any history of conditions that could predispose him to an increased risk of bruising. The purpura resolved after 6 days.

### Case 2

**Fig 2:**
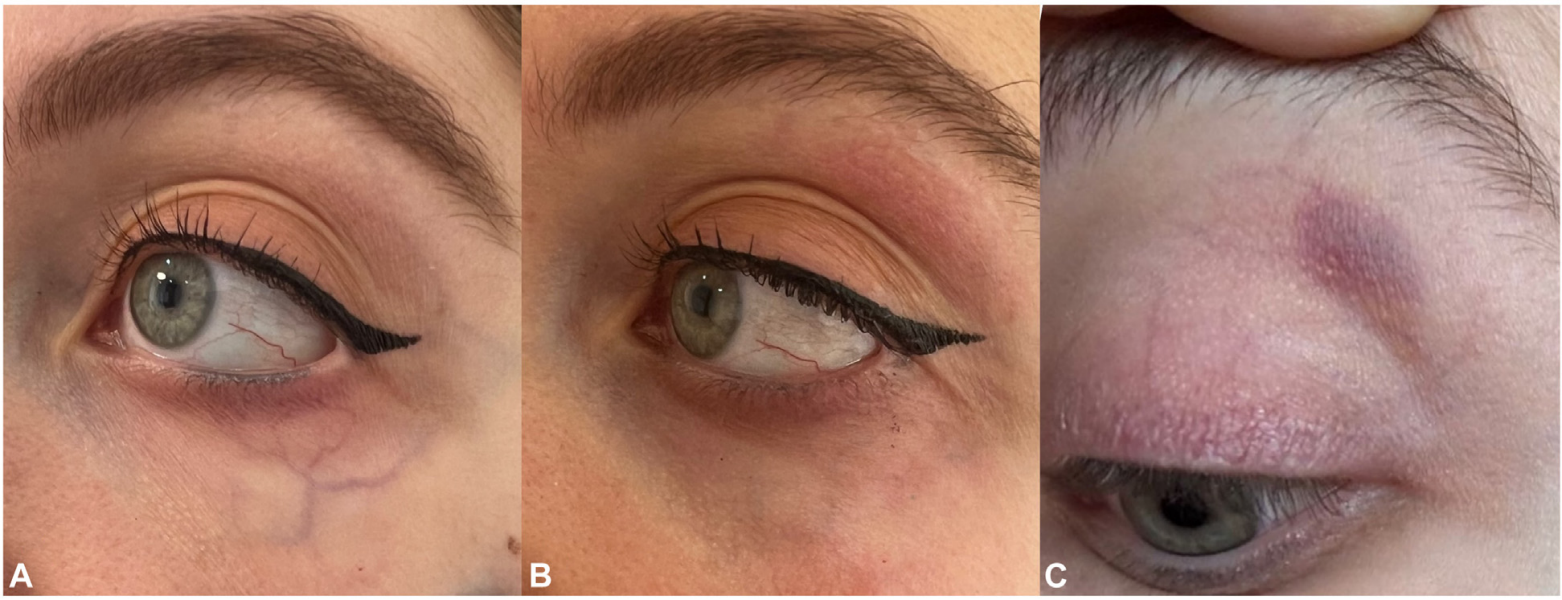
**A,** Before, (**B**) Immediately after, and (**C**) 1 month after the laser procedure.

A 32-year-old Lebanese female experienced bruising in her left upper eyelid 1 month after the procedure (Fig 2). She denied any medications/supplements or any history of conditions that could predispose her to an increased risk of bruising. The purpura resolved after 7 days.

### Case 3

**Fig 3:**
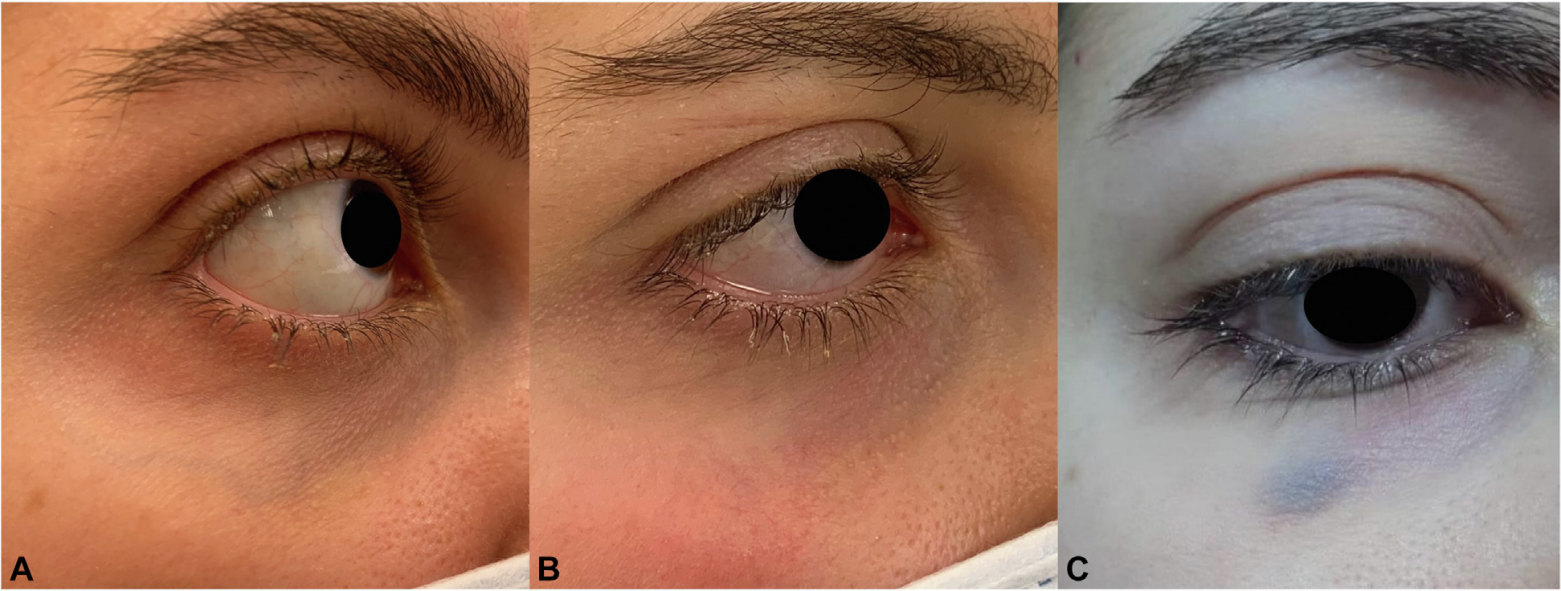
**A,** Before, (**B**) Immediately after, and (**C**) 1 month after the laser procedure.

A 27-year-old Lebanese female developed bruising in her right lower eyelid 1 month after the procedure (Fig 3). She was not taking any medications or supplements and had no history of conditions that might increase her risk of bruising. The purpura resolved within 7 days.

### Case 4

A 35-year-old Lebanese female developed bruising in her left lower eyelid 1.5 month after the procedure. She had no medications, supplements, or underlying conditions that could predispose her to an increased risk of bruising. The purpura resolved within a few days.

## Discussion

The 1064 nm Nd:YAG laser is the most commonly used laser to treat periorbital vessels. While this wavelength has a lower absorption coefficient for hemoglobulin, deeper penetration into the skin makes it suitable for deeper venous lesions. The diode and alexandrite lasers are also effective, but are less commonly used due to significantly higher melanin absorption which increases the risk of retinal damage.^7^^,^^8^ Periorbital vessels are typically treated with long millisecond pulse durations with a clinical endpoint of vessel disappearance or temporary graying/darkening.^6^ Theoretically, this pulse duration should not cause purpura, although this can rarely occur immediately post-treatment. More common side effects include transient discomfort, erythema, and edema. Rare complications include necrosis, burns, hyperpigmentation, and hypopigmentation.

To our knowledge, this case series is the first to document a previously unreported adverse event of delayed onset purpura following treatment of periorbital veins with a 1064 nm Nd:YAG laser. Interestingly, in our case series this adverse event was observed with both cryogen spray and chilled laser tip methods of skin cooling. While the precise mechanism underlying this observed adverse event remains unclear, we hypothesize that treatment induces transient vascular remodeling and fragility, predisposing the area to purpura from routine activities such as rubbing or massaging the treated region. While these actions wouldn't typically affect untreated skin, they could cause purpura in treated areas still recovering from laser treatment. Obtaining histological samples would be challenging and invasive in the periocular area. This limitation highlights the need for further research to validate the proposed mechanism.

A study by Li et al demonstrated that the 1064 nm laser primarily induces vessel constriction immediately post-treatment.^9^ However, the study also observed varied responses, including hemorrhage and re-expansion of vessels within 1 hour after treatment. While these variable responses were studied only in the short term, they suggest variability in the post-treatment healing response, potentially rendering vessels more fragile during this period. This supports our hypothesis regarding the increased susceptibility of treated vessels to adverse events in the weeks following treatment.

Moreover, both theoretical models and clinical studies suggest that microvascular remodeling, including changes in vessel diameter and blood flow redistribution, may persist for weeks following laser vessel ablation, with some long-term effects, such as vessel recurrence or evolving changes, documented up to several months post-treatment.^10^, ^11^, ^12^

Given the transient and potentially underreported nature of this adverse event, we emphasize the importance of patient education. Patients should be advised to exercise caution while handling the treated area for several months post-procedure.

## Conflicts of interest

None disclosed.
